# Differential effects of tyrosine-rich amelogenin peptide on chondrogenic and osteogenic differentiation of adult chondrocytes

**DOI:** 10.1007/s00441-015-2292-7

**Published:** 2015-09-25

**Authors:** H. D. Amin, C. R. Ethier

**Affiliations:** Department of Bioengineering, Imperial College London, London, UK; Wallace H. Coulter Department of Biomedical Engineering, Georgia Institute of Technology & Emory University School of Medicine, Atlanta, Georgia USA

**Keywords:** Cartilage repair, ACI, Chondrogenesis, Amelogenin, Stem cells

## Abstract

Current approaches to treat osteoarthritis (OA) are insufficient. Autologous chondrocyte implantation (ACI) has been used for the past decade to treat patients with OA or focal cartilage defects. However, a number of complications have been reported post-ACI, including athrofibrosis and symptomatic hypertrophy. Thus, a long-term ACI strategy should ideally incorporate methods to ‘prime’ autologous chondrocytes to form a cartilage-specific matrix and suppress hypertrophic mineralization. The objective of this study is to examine the effects of tyrosine-rich amelogenin peptide (TRAP; an isoform of the developmental protein amelogenin) on human articular cartilage cell (HAC) chondrogenic differentiation and hypertrophic mineralization in vitro. Effects of chemically synthesized TRAP on HAC chondrogenic differentiation were determined by assessing: (1) sGAG production; (2) Alcian blue staining for proteoglycans; (3) collagen type II immunostaining; and (4) expression of the chondrogenic genes *SOX9*, *ACAN* and *COL2A1*. Hypertrophic mineralization was assayed by: (1) ALP expression; (2) Alizarin red staining for Ca^+2^-rich bone nodules; (3) OC immunostaining; and (4) expression of the osteogenic/hypertrophic genes *Ihh* and *BSP*. Chemically synthesized TRAP was found to suppress terminal osteogenic differentiation of HACs cultured in hypertrophic mineralization-like conditions, an effect mediated via down-regulation of the *Ihh* gene. Moreover, TRAP was found to augment chondrogenic differentiation of HACs via induction of *SOX9* gene expression when cells were cultured in pro-chondrogenic media. The results obtained from this proof-of-concept study motivate further studies on the use of TRAP as part of a preconditioning regimen in autologous chondrocyte implantation procedures for OA patients and patients suffering from focal cartilage defects.

**Electronic supplementary material:**

The online version of this article (doi:10.1007/s00441-015-2292-7) contains supplementary material, which is available to authorized users.

## Introduction

Osteoarthritis (OA) is a joint disease characterized by progressive degeneration of articular cartilage (Felson 2006). It was recently identified as the second most common cause of disability and the major cause for disability allowance in the United States (Felson 2006). OA is associated with the loss of the proteoglycan-rich extracellular matrix (ECM) of cartilage and subsequent ECM mineralization (Felson 2006). Physiotherapy, exercise and analgesics are currently used for early stage management of OA, whereas in later stages, surgical interventions are typically required (Bartha, et al. 2006; Felson 2006). However, existing therapies do not reverse the development of OA and achieve cartilage regeneration (Bartha et al. 2006; Felson 2006).

Current efforts to develop regenerative medicine-based therapeutic interventions for OA or focal cartilage defect repair include the use of autologous chondrocyte implantation (ACI), often together with a polymer-based three-dimensional scaffold matrix (Clouet et al. 2009; Getgood et al. 2009). In ACI, arthroscopically isolated cartilage cells from an undamaged cartilage region are cultured in vitro to scale-up cell numbers and seeded into scaffolds that are then implanted in cartilage defects. In addition to autologous chondrocytes, other progenitor cells (i.e., bone marrow stromal cells, BMSCs and umbilical cord stromal cells) have also been widely used in such constructs (Clouet et al. 2009; Getgood et al. 2009; Temenoff and Mikos 2000). Unfortunately, these tissue-engineering (TE) approaches have not provided long-term clinical benefit for patients suffering from OA or focal cartilage defects (Brittberg et al. 1994; Clouet et al. 2009; Getgood et al. 2009; Lyngstadaas et al. 2009; Niemeyer et al. 2008; Peterson et al. 2000; Temenoff and Mikos 2000). For example, the occurrence of subsequent surgical procedures (SSPs) is common following ACI (Phillip-Niemeyer et al. 2008), e.g., in one trial, 49 % of patients receiving ACI subsequently underwent an SSP on the treated knee within 4 years of initial treatment (Phillip-Niemeyer et al. 2008). Moreover, symptomatic hypertrophy was observed following ACI for patellar defects (Phillip-Niemeyer et al. 2008).

In view of the above, there would be great value in developing a therapy that could precondition and ‘prime’ autologous articular cartilage cells to produce hyaline cartilage-associated ECM as well as suppress any aberrant hypertrophic mineralization in ACI. We suggest that a novel molecule related to the enamel matrix derivative (EMD) may be useful in this regard. EMD is a complex mixture of developmental proteins (amelogenin and its proteolytic clipping forms/splicing forms) and plays a pivotal role in craniofacial tissue development (Lyngstadaas et al. 2009). We have recently shown that EMD can modulate various differentiation pathways in adult ligament precursor cells in vitro, including osteogenic, adipogenic and chondrogenic (Amin et al. 2012, 2013). We consider here tyrosine-rich amelogenin peptide (TRAP), a 5.1-kDa isoform of EMD generated from a full-length amelogenin through proteolytic clipping. Both naturally occurring and chemically synthesized forms of TRAP have recently been shown to suppress bone-forming activity in various osteogenic precursor cells (i.e., adult ligament cells and alveolar bone cells (Amin et al. 2012)), although the effects of this peptide on chondrogenic differentiation and hypertrophic mineralization have not hitherto been examined.

In view of TRAP’s ability to down-regulate osteogenic differentiation (Amin et al. 2012), we hypothesized that TRAP acts selectively on the mesenchymal lineage commitment pathways. The present study therefore examines the effects of chemically synthesized TRAP on chondrogenic differentiation and hypertrophic mineralization of healthy human chondrocytes in vitro.

## Materials and methods

### Cell culture

Ostensibly normal adult primary human articular cartilage cells (HACs) from three male donors (ages 20–40 years; purchased at passage 0 from Asterand, Roystone, UK) were used in the present study. HACs were cultured in growth media (GM) at 37 °C in a humidified atmosphere of 5 % CO_2_ in air, as described previously (Amin et al. 2012, 2013, 2014). Cells were scaled up in multilayer tissue culture flasks until 80–90 % confluent, followed by experiments in pellet cultures between passages 1 and 3. Detailed descriptions of the culture media are given in the Supplementary material.

We chose to use HACs, rather than BMSCs, for the present study determining the effects of TRAP on hypertrophic differentiation for several reasons. First, bone marrow has a highly heterogeneous stromal cell population, containing stem/progenitor cells with the ability to undergo multi-lineage differentiation, osteogenic progenitor cells and osteoblasts. These osteogenic progenitor cells/osteoblasts can readily differentiate and form bone-like mineralized nodules, confounding the present study of hypertrophic mineralization, i.e., of the endochondral ossification pathway where mature chondrocytes undergo hypertrophy to form bone cells. Second, the use of HACs better replicates the clinical scenario of ACI, where cells harvested from existing cartilage are used.

### Treatment of cells with TRAP

Chemically synthesized 45-amino acid TRAP (sequence: NH_2_-MPLPPHPGHPGYINFSYEVLTPLKWYQNMIRHPYTSYGYEPMGGW–COOH; purchased from Insight Biotech, London, UK) was diluted in cell culture grade water (Thermo Scientific, Basingstoke, UK) and added directly to the culture media with cell pellets to yield a final concentration of 1–100 μg/ml, previously shown to be within the solubility limit for this form of TRAP (Amin et al. 2015).

### Effects of TRAP on sGAG and ALP production of HACs under chondrogenic conditions

Based on previous studies (Amin et al. 2012, 2013), we examined the effects of 1, 10, 50 and 100 μg/ml TRAP on terminal chondrogenic differentiation of HACs cultured as pellets, as described previously (Amin et al. 2014). Briefly, cells were scaled up in GM, trypsinized and cultured (0.5 × 10^6^ cells/pellet in 15-ml conical polypropylene Falcon tubes with caps loosened to allow gas exchange (Nunc)) in GM overnight to obtain cell pellets. Culture media was then replaced by fresh GM or chondrogenic media (CM), as described previously (Amin et al. 2014). Cells cultured in non-lineage-specific GM was used here as controls. Pellets were then cultured for 3 weeks in the presence or absence of TRAP. After 3 weeks, chondrogenic differentiation was evaluated by quantifying sulfated glycosaminoglycan (sGAG) content in pellets (Blyscan kit; Biocolor, Carrickfergus, UK). ALP was measured using a p-nitrophenyl phosphate kit (Sigma) in a separate set of HAC pellets. Total DNA content was quantified using the PicoGreen^®^ double-stranded DNA assay (Invitrogen). All sGAG and ALP amounts were normalized by DNA content. sGAG, ALP and DNA quantification assays were carried out as per instructions from the manufacturers and as previously described (Amin et al. 2012, 2014).

### Effects of TRAP on sGAG and ALP production of HACs under hypertrophic conditions

Cell pellets were first cultured in CM for 2 weeks (“preconditioned”) in order to obtain functionally mature chondrocytes (based on their ability to produce sGAG), as described above. After 2 weeks of incubation with CM, these pellets were further cultured in hypertrophic media (HTM) alone or HTM + TRAP for 3 weeks, after which pellets underwent sGAG, ALP and DNA quantification, as described above. Cells cultured in non-lineage specific GM was used here as controls.

Methods for histology, immunocytochemistry and gene expression analyses are described in Supplementary materials.

## Results

HACs (previously shown to contain a ‘stem’/progenitor cell-like population (Williams et al. 2010)) were grown in pellet culture and were exposed to a variety of conditions with or without TRAP. Specifically, growth media (GM) was employed to promote cell division, chondrogenic media (CM) to promote chondrogenesis or hypertrophic media (HTM) to promote hypertrophic mineralization.

### In vitro functional outcomes: Effects of TRAP on differentiation of articular cartilage cells

In TRAP-treated cells cultured in CM, a large dose-dependent increase in sGAG levels was observed (Table 1); specifically, 10 and 50 μg/ml TRAP increased sGAG levels by 353 and 344 %, respectively, compared with control conditions of HACs cultured in CM alone (*p* < 0.05). Further, TRAP produced no significant change in alkaline phosphatase (ALP) activity, a key functional indicator of terminal osteogenic differentiation in vitro (Table 1). HACs cultured in GM in the presence of TRAP (1–100 μg/ml) did not exhibit any change in sGAG production or ALP activity (data not shown).

**Table 1:** Effects of various TRAP concentrations (1–100 μg/ml) on sGAG and ALP production by HACs

	Growth media	Chondrogenic media				
		0 μg/ml TRAP (control)	1 μg/ml TRAP	10 μg/ml TRAP	50 μg/ml TRAP	100 μg/ml TRAP
Relative sGAG/DNA (μg/μg)	GM (3 weeks)	CM (3 weeks)				
	1	3.6 ± 1.1*	7.3 ± 1.5*†	12.7 ± 2.1*†	12.4 ± 2.0*†	8.3 ± 1.4*
	GM (5 weeks)	CM (2 weeks), followed by HTM (3 weeks)				
	1	1.6 ± 1.1	3.4 ± 1.2*	6.7 ± 1.1*§	5.2 ± 1.2*§	4.7 ± 1.4*§
Relative ALP\DNA (μg/μg)	GM (3 weeks)	CM (3 weeks)				
	1	1.9 ± 0.5*	1.9 ± 0.5*	1.8 ± 0.6	1.6 ± 0.5	1.7 ± 0.5
	GM (5 weeks)	CM (2 weeks), followed by HTM (3 weeks)				
	1	4.4 ± 0.7*	1.4 ± 0.2§	1.6 ± 0.4§	0.9 ± 0.1§	0.9 ± 2§

Row 2: sGAG levels assayed in cell pellets cultured in growth media (*GM*), chondrogenic media (*CM*) or CM + TRAP for 3 weeks. Row 4: sGAG levels assayed in cell pellets preconditioned in CM for 2 weeks followed by 3 weeks of culture in hypertrophic media (*HTM*) or HTM + TRAP, for which 5 weeks of culture in GM was used as a baseline. Rows 6 and 8: similar to rows 2 and 4, except that ALP levels are shown. The tabulated quantities are the relative sGAG/DNA ± SE and relative ALP/DNA ± SE ratios, all normalized to values from cells with GM

†*p* < 0.05 compared with CGM; *p < 0.05 compared with CM alone; §*p* < 0.05 compared with HTM alone

For determining the effects of TRAP on hypertrophic mineralization, HACs were first preconditioned for 2 weeks in CM and then cultured for 3 weeks in HTM or HTM + TRAP, as a simplified in vitro model of conditions changing from chondrogenic to osteogenic in a process of endochondral ossification in osteoarthritic joints. Notably, cells cultured in the presence of TRAP exhibited suppressed ALP activity (Table 1) compared to HACs cultured without TRAP (*p* < 0.05). Interestingly, this effect was not dose-dependent, as 1–100 μg/ml TRAP concentrations showed broadly similar effects.

Further, the results also showed that, at all concentrations tested, TRAP-treated HACs cultured in HTM had sGAG levels close to those of HACs grown in CM, while cell pellets grown in HTM without TRAP had minimal sGAG levels (*p* < 0.05). Specifically, 10 and 50 μg/ml TRAP increased sGAG levels by 418 and 325 %, respectively, compared with cells grown without TRAP (*p* < 0.05), with a maximal effect at 10 μg/ml TRAP (Table 1).

Together, these data demonstrate that, when HACs are grown under hypertrophic conditions, TRAP suppresses terminal hypertrophic mineralization while maintaining sGAG levels similar to those found in CM alone. Since 10 μg/ml TRAP had the most pronounced effects, this concentration was used for subsequent experiments.

### In vitro terminal differentiation outcomes: Effects of TRAP on HAC chondrogenic and osteogenic differentiation

**Fig. 1:**
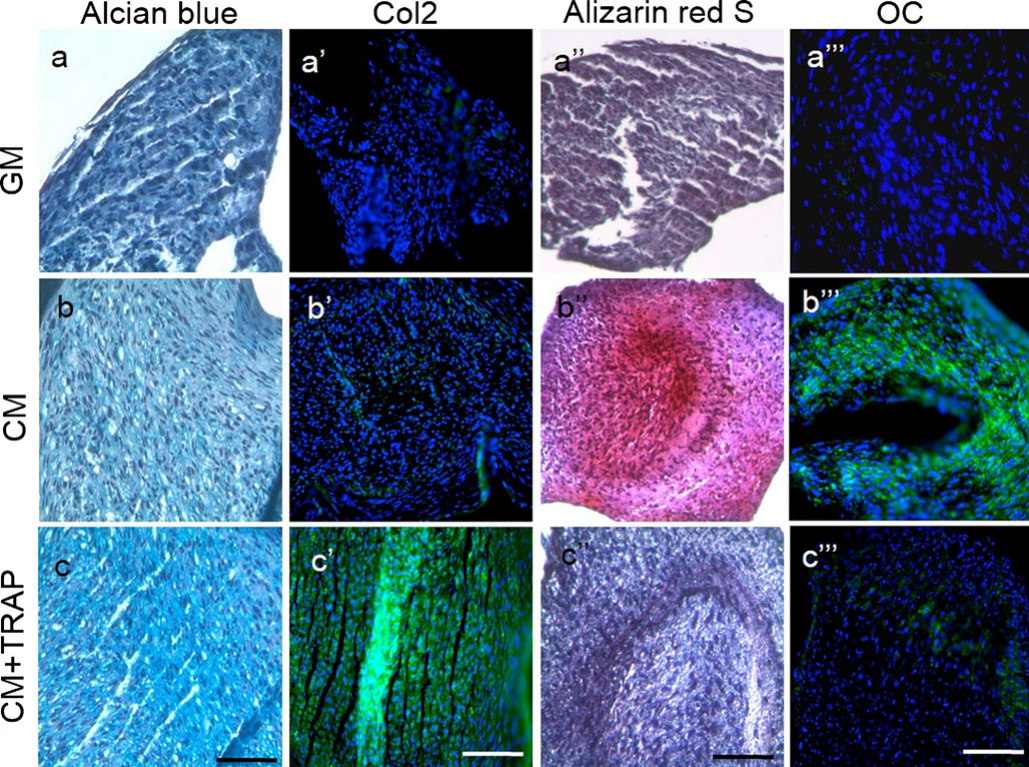
Histology indicates that 10 μg/ml TRAP promotes chondrogenic differentiation and suppresses hypertrophic mineralization. *Labels* on individual *panels* refer to culture media type: **a**–**a”’** GM; **b**–**b”’** CM; **c**–**c”’** CM + TRAP. Note the intense bright blue staining of TRAP-treated cell pellets stained with Alcian blue (**c**; indicative of glycosaminoglycans present in the ECM) and the corresponding lack of Alizarin red staining (**c”**; indicative of minimal calcium deposition). Note also the green fluorescent staining of TRAP-treated cell pellets immuno-stained with Col2 (**c’**) and the corresponding lack of OC staining (**c”’**). Alcian blue and Alizarin red sections were counter-stained with Harris Haematoxylin (purple nuclei); Col2 and OC sections were counter-stained with Hoechst dye (blue nuclei).* Scale bar* 100 μm

HAC pellets cultured for 3 weeks in CM with 10 μg/ml TRAP showed strong Alcian blue staining (Fig. 1c), indicating abundant ECM proteoglycans and glycosaminoglycans. In contrast, control cultures grown in GM or CM without TRAP showed weaker staining (Fig. 1a, b). A dark, purple-colored background staining of pellets grown in GM was also observed, compared with the blue-colored matrix in CM and CM + TRAP. The purple staining of pellets grown in GM may be due to the residual non-specific background staining of Harris hematoxylin. Additionally, strong Col2 staining in the ECM of HAC pellets cultured in CM + TRAP was observed, compared with relatively low levels of Col2 staining in cultures grown in CM and little if any staining in cultures grown in GM (Fig. 1a’–c’).

Further, HAC pellets preconditioned in CM for 2 weeks followed by 3 weeks of culture in HTM alone exhibited strong Alizarin red staining of Ca^+2^-rich bone-like nodules; cells cultured in GM showed little or no staining (Fig. 1b”). Notably, 10 μg/ml TRAP completely abolished staining for bone-like nodules in HAC pellets cultured in HTM (Fig. 1c”). Further, strong osteocalcin (OC) staining in the ECM of HAC pellets was observed when cultured in HTM alone, while little or no staining was observed for cells cultured in GM (Fig. 1a”’, b”’). In contrast, TRAP completely abrogated OC staining in HAC pellets cultured in HTM (Fig. 1c”’). Thus, histology confirms that TRAP suppresses terminal osteogenic differentiation of HACs cultured under hypertrophic mineralization-inducing conditions.

### Gene expression analyses: Effects of TRAP on HAC chondrogenic and osteogenic genes

**Fig. 2:**
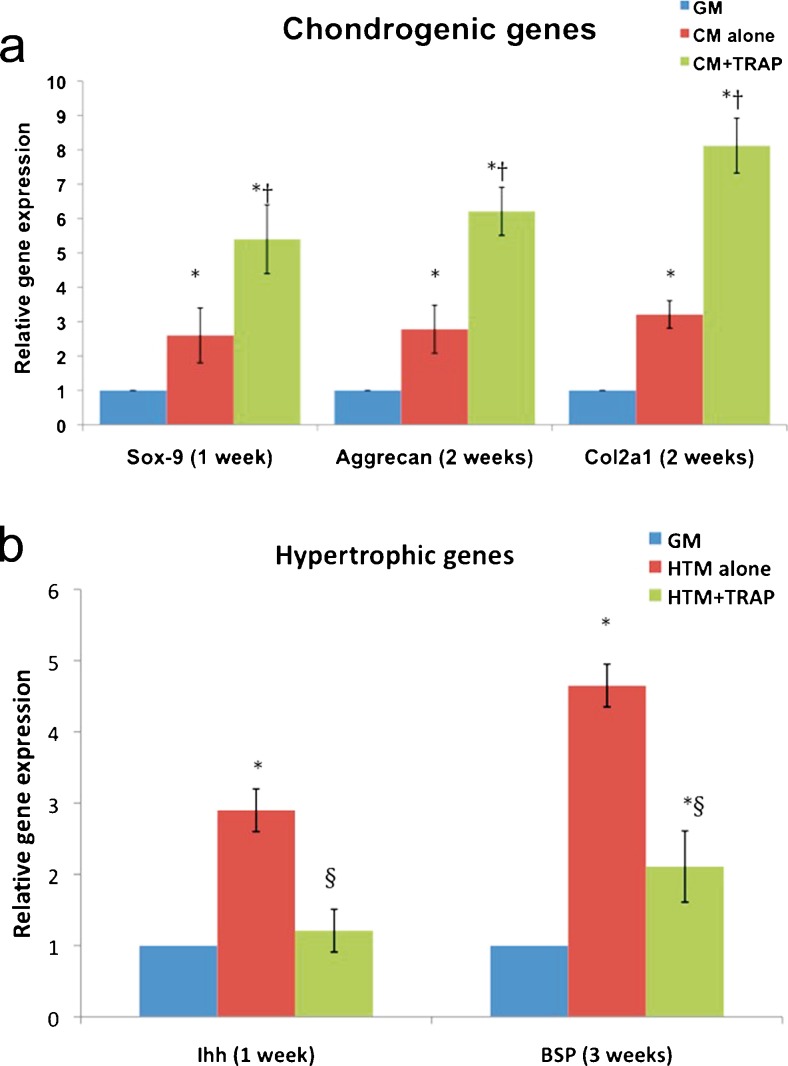
Ten μg/ml TRAP enhances mRNA levels of pro-chondrogenic genes and suppresses levels of hypertrophic mineralization genes. **a** Note the up-regulation of the early chondrogenic gene *SOX9* and the late genes *ACAN* and *COL2A1* of HAC pellets treated with TRAP. **b** Note the down-regulation of the early hypertrophic mineralization gene *Ihh* and the late bone gene *BSP* of TRAP-treated HAC pellets. Plotted values are the means ± SE of triplicate measurements, as described in the text. *p < 0.05 compared with GM; *†*p* < 0.05 compared with CM alone; or *§*p* < 0.05 compared with HTM alone

Results showed that cells cultured in CM + 10 μg/ml TRAP had significantly higher levels of *SOX9* (2.0-fold), *ACAN* (2.2-fold) and *COL2A1* (2.5-fold) transcripts, compared with cells cultured in CM alone (*p* < 0.05). As expected, HAC pellets cultured in CM and in CM + TRAP expressed higher levels of the early chondrogenic transcription factor *SOX9* and the late genes *ACAN* and *COL2A1*, compared with pellet cultures in GM (*p* < 0.05) (Fig. 2a), suggesting that TRAP induces both early and late chondrogenic genes in HACs.

As expected, HAC pellets preconditioned in CM for 2 weeks followed by 1 week of culture in HTM alone expressed higher levels of the early osteogenic/hypertrophic marker *Ihh* and the late bone matrix gene *BSP*, compared with pellet cultures in GM (Fig. 2b). However, cells cultured in HTM + TRAP had significantly reduced transcript levels of *Ihh* (59 % reduction; *p* < 0.05) and *BSP* (55 % reduction; *p* < 0.05), compared with pellets cultured in HTM alone (Fig. 2b). These results indicate that TRAP suppresses both early and late pro-hypertrophic mineralization genes.

## Discussion

Our results show that TRAP, a chemically synthesized amelogenin protein isoform, suppresses hypertrophic mineralization and concomitantly promotes chondrogenic differentiation of HACs. This result is supported by data on expression levels of both early and late pro-chondrogenic and pro-hypertrophic mineralization genes, as well as by data at the protein level. We note that the above gene expression study was carried out using a conventional semi-quantitative RT-PCR; use of a quantitative gene expression assay technique (quantitative-real-time PCR) would be beneficial for future studies.

*SOX9* up-regulation is a pivotal upstream chondrogenic event in the up-regulation of *COL2A1*, *ACAN* and in sGAG production (Bi et al. 1999), while *RUNX2* up-regulation is a key upstream osteogenic event in the up-regulation of OC, BSP and in ALP production (Kim et al. 2013). In view of our previous report showing that TRAP suppresses bone-forming activity through *Smad6*-mediated *RUNX2* inhibition (Amin et al. 2012), it is possible that TRAP may be differentially regulating chondrogenic and osteogenic activity of adult precursor cells by regulating activities of the ‘key’ lineage-specific early transcription factors *SOX9 and RUNX2* (Bi et al. 1999; Kim et al. 2013). This hypothesis requires further investigation.

The pro-chondrogenic effects of 10 μg/ml TRAP were observed when cells were cultured in full chondrogenic conditions (an environment facilitating chondrogenesis) but not in non-selective growth conditions. This suggests that an environment favoring chondrogenesis, e.g., one in which TGF-β3 is present, may be essential for TRAP-mediated chondrogenesis. In future studies, it might therefore be important to identify molecular mechanisms involved in TRAP-induced chondrogenesis in order to understand this synergy between TRAP and chondrogenic media components.

As noted above, TRAP is a proteolytic product of full-length amelogenin and is shown to be secreted as part of a heterogeneous mixture of EMP during craniofacial developmental (Lyngstadaas et al. 2009). In the natural form of TRAP, the SER16 amino acid has been shown to be phosphorylated (Lyngstadaas et al. 2009). Although the present study did not examine the effects of phospho-SER16 TRAP on osteogenic and chondrogenic differentiation, we have shown in our previous studies that both naturally occurring phospho-SER16 TRAP and chemically synthesized non-phosphorylated TRAP exhibited the same inhibitory effects on osteogenic differentiation (Amin et al. 2012). Thus, the present study focused on examining the effects of the non-phosphorylated synthetic form of TRAP on osteogenic and chondrogenic differentiation of chondrocytes. TE strategies for cartilage replacement, based on autologous chondrocytes and MSCs, have recently received significant attention (Amin et al. 2014; Getgood et al. 2009). However, any long-term cartilage repair strategy based on ACI must incorporate methods to prime autologous chondrocytes for cartilage-specific matrix production while suppressing the hypertrophic mineralization pathway, so as to prevent athrofibrosis, joint adhesion, cartilage injury and symptomatic hypertrophy post-ACI (Brittberg et al. 1994; Dreier 2010; Goldring and Goldring 2010; Niemeyer et al. 2008; Peterson et al. 2000). Although we recognize that this is a cell culture-based study, with the limitations implied thereby, our results motivate further investigation into the use of TRAP for the above purpose. Specifically, future experiments/investigations should examine three specific points: (1) determine the molecular mechanism(s) involved in TRAP-mediated chondrogenic differentiation and suppression of hypertrophic bone formation; (2) identify and characterize an articular cartilage cellular subset (if present) that specifically reacts to the TRAP signal(s); and (3) ultra-structurally compare TRAP-stimulated cartilage to pristine cartilage and fibrocartilage.

If further studies with TRAP are promising, there may be a number of advantages to the eventual clinical use of TRAP: (1) due to its small size (45 amino acids), the chemically synthesized peptide should be relatively inexpensive to produce in substantial quantities with high purity; (2) TRAP can potentially increase the effectiveness of ACI procedures, both for OA patients and for patients with focal cartilage defects due to sports (or other) injuries; and (3) incorporation of a cell preconditioning step into a well-established, routine ACI protocol would be relatively easy. We hope that this approach can eventually delay/reduce the need for expensive surgical procedures involving prosthetic implants and increase the quality of life for patients suffering from joint defects.

## Electronic supplementary material

Below is the link to the electronic supplementary material.

ESM 1 (DOCX 29 kb)

## References

[CR1] Amin HD, Olsen I, Knowles JC, Donos N (2012). Differential effect of amelogenin peptides on osteogenic differentiation in vitro: identification of possible new drugs for bone repair and regeneration. Tissue Eng A.

[CR2] Amin HD, Olsen I, Knowles JC, Dard M, Donos N (2013). Effects of enamel matrix proteins on multi-lineage differentiation of periodontal ligament cells in vitro. Acta Biomater.

[CR3] Amin HD, Brady MA, St-Pierre JP, Stevens MM, Overby DR, Ethier CR (2014) Stimulation of chondrogenic differentiation of adult human bone marrow-derived stromal cells by a moderate-strength static magnetic field. Tissue Eng Part A 20:1612–1620 10.1089/ten.tea.2013.0307 PMC4029136 24506272

[CR4] Amin HD, Olsen I, Knowles JC, Dard M, Donos N (2015). Interaction of enamel matrix proteins with human periodontal ligament cells. Clin Oral Invest.

[CR5] Bartha L, Vajda A, Duska Z, Rahmeh H, Hangody L (2006). Autologous osteochondral mosaicplasty grafting. J Orthopaedic Sports Phys Ther.

[CR6] Bi W, Deng JM, Zhang Z, Behringer RR, de Crombrugghe B (1999). Sox9 is required for cartilage formation. Nat Genet.

[CR7] Brittberg M, Lindahl A, Nilsson A, Ohlsson C, Isaksson O, Peterson L (1994). Treatment of deep cartilage defects in the knee with autologous chondrocyte transplantation. N Engl J Med.

[CR8] Clouet J, Vinatier C, Merceron C, Pot-vaucel M, Maugars Y, Weiss P, Grimandi G, Guicheux J (2009). From osteoarthritis treatments to future regenerative therapies for cartilage. Drug Discov Today.

[CR9] Dreier R (2010). Hypertrophic differentiation of chondrocytes in osteoarthritis: the developmental aspect of degenerative joint disorders. Arthritis Res Therapy.

[CR10] Felson DT (2006). Osteoarthritis of the knee. N Engl J Med.

[CR11] Getgood A, Bhullar T, Rushton N (2009). Current concepts in articular cartilage repair. Orthopaedics Trauma.

[CR12] Goldring MB, Goldring SR (2010). Articular cartilage and subchondral bone in the pathogenesis of osteoarthritis. Ann N Y Acad Sci.

[CR13] Kim EJ, Cho SW, Shin JO, Lee MJ, Kim KS, Jung HS (2013). Ihh and Runx2/Runx3 signaling interact to coordinate early chondrogenesis: a mouse model. PLoS ONE.

[CR14] Lyngstadaas SP, Wohlfahrt JC, Brookes SJ, Paine ML, Snead ML, Reseland JE (2009). Enamel matrix proteins; old molecules for new applications. Orthodontics Craniofacial Res.

[CR15] Niemeyer P, Pestka JM, Kreuz PC, Erggelet C, Schmal H, Suedkamp NP, Steinwachs M (2008). Characteristic complications after autologous chondrocyte implantation for cartilage defects of the knee joint. Am J Sports Med.

[CR16] Peterson L, Minas T, Brittberg M, Nilsson A, Sjögren-Jansson E, Lindahl A (2000). Two-to 9-year outcome after autologous chondrocyte transplantation of the knee. Clin Orthop Relat Res.

[CR17] Temenoff JS, Mikos AG (2000). Review: tissue engineering for regeneration of articular cartilage. Biomaterials.

[CR18] Williams R, Khan IM, Richardson K, Nelson L, McCarthy HE, Analbelsi T (2010). Identification and clonal characterization of a progenitor cell sub-population in normal human articular cartilage. PLoS ONE.

