# Supplementary material for: Differential effects of tyrosine-rich amelogenin peptide on chondrogenic and osteogenic differentiation of adult chondrocytes
Source: Cell Tissue Res. 2015 Sep 25;364:219–24. doi: 10.1007/s00441-015-2292-7 (PMC4819750; doi:10.1007/s00441-015-2292-7)
Supplement: Supplementary file 1 — (DOCX 29 kb) [file 441_2015_2292_MOESM1_ESM.docx]

**Supplementary material**

**Methods**

**Reagents and culture media compositions**

We used growth media (GM), containing alpha-minimum essential media (α-MEM) (Invitrogen, Paisley, UK), 10% foetal bovine serum (Invitrogen), 5 ng/ml basic fibroblast growth factor (bFGF) (Peptrotech, London, UK) and 1% p/s (Sigma, Dorset, UK), to scale up human articular cartilage cells. Chondrogenic media (CM), containing DMEM-high glucose (Invitrogen), 40 µg/ml L-proline, 50 µg/ml ascorbate-2-phosphate, 100 nM dexamethasone, ITS and 1% penicillin/streptomycin (p/s) (all from Sigma) and 10 ng/ml TGFβ3 (Peprotech) was used as selective culture media to promote HAC chondrogenic differentiation, as described previously (Amin, et al., 2014). Hypertrophic media (HTM), containing DMEM-high glucose, 1% p/s, 1xITS, 50 μg/ml ascorbic acid, 50 ng/ml thyroxin, 20 mM β-glycerophosphate and 1 nM dexamethasone (all from Sigma) was used to promote hypertrophic mineralization of HACs, as described in (Mackay, et al., 1998).

**Alizarin red and Alcian blue staining**

For chondrogenic differentiation, HAC pellets were cultured for 3 weeks in GM, CM, or CM+TRAP, while for hypertrophic mineralization pellets were cultured for a total of 5 weeks in GM, or for 2 weeks in CM followed by 3 weeks in HTM or HTM+TRAP, as described in Methods. After 3 or 5 weeks, Alcian blue or Alizarin red staining for proteoglycans and calcium-rich bone-like nodules, respectively, was carried out as described previously (Amin, et al., 2012, Amin, et al., 2014). Paraffin sections were also immunolabelled for Col2 and osteocalcin (OC), as described previously Amin, et al., 2012, Amin, et al., 2014).

Cell pellets were fixed in 4% paraformaldehyde at 4˚C for 24 h, dehydrated in an ascending series of ethanols (40, 70, 90 and 100% ethanol; 20 min/step) and embedded in paraffin, as described previously (Clouet, et al., 2009). 3 µm sections were cut and stained with 1% Alcian blue (pH 2.5) (Sigma) for 5 min. For Alizarin red staining, sections were stained with 2% Alizarin red S (pH 4.2) for 30 min. Nuclei were counterstained using Harris haematoxylin.

**Immunocytochemistry**

Paraffin sections were also immunolabelled for Col2 and osteocalcin (OC), as described previously (Amin, et al., 2012, Amin, et al., 2014). Briefly, tissue in sections was treated with 1 mg/ml pepsin (in 10 mM Tris-HCl, pH 2.0) (Sigma) for 15 min at room temperature to retrieve antigen sites and then permeabilized using 0.1% Triton X (Sigma) for 15 min at room temperature (RT), followed by 1 h incubation at RT with a blocking solution containing 10% normal goat serum (NGS) (Life Technologies) in phosphate-buffered saline (PBS). Sections were then incubated for 2 h at RT with primary mouse monoclonal anti-Col2 or mouse monoclonal anti-OC (Abcam, Cambridge, UK) diluted 1:100 in PBS containing 1% NGS. Incubation was then carried out with goat anti-mouse Alexa Fluor secondary antibody (Life technologies) diluted 1:200 in PBS containing 1% NGS for 1 h at RT. Col2- and OC-positive cells were visualized by their green fluorescent cytoplasmic and ECM staining. Nuclei were counterstained using Hoechst dye (Life Technologies).

**Gene expression**

Total RNA was extracted from replicate pellet cultures (cultured in GM, CM or CM+TRAP) for RT-PCR analysis of the early chondrogenic transcription factor *SOX9* (measured after 1 week of culture) and the late marker genes *COL2A1* and *ACAN* (measured after 2 weeks of culture), as described previously (Amin, et al., 2014). Since mRNA transcripts are expressed much earlier than the corresponding proteins, the *COL2A1* gene was measured at week 2 and immunostaining of Col2 protein was carried out at week 3 (as described above). For hypertrophic mineralization gene expression analysis, total RNA was extracted from replicate pellet cultures (cultured in GM, HTM or HTM+TRAP) for the early hypertrophic marker gene *Ihh* (1 week post-preconditioning) and the late marker gene *BSP* (3 weeks post-preconditioning). Glyceraldehyde 3-phosphate dehydrogenase (*GAPDH*), a housekeeping gene, was used as an endogenous control.

The sequences of the forward and reverse primers (from Sigma) were: *SOX9* forward: ATCTGAAGAAGGAGAGCGAG, reverse: TCAGAAGTCTCCAGAGCTTG; *COL2A1* forward: TTTCCCAGGTCAAGATGGTC, reverse: CTTCAGCACCTGTC CACCA; *ACAN* forward: TGAGGAGGGCTGGAACAAGTACC, reverse: GGAGGTGGTAATTGCAGGGAACA; *Ihh* forward: GAGGAGTCCCTGCATTATGA, reverse: CAGGAAAATGAGCACATCGC; *BSP* forward: TGCTCAGCATTTTGGGAAT, reverse: TGCATTGGCTCCAGTGACACT; *GAPDH* forward: CCACCCATGGCAAATTCCCATGGCA, reverse: CTGGACGGCAGGTCAGGTCCACC. *SOX9*, *COL2A1* and *ACAN* primers were used at annealing temperatures of 58, 58 and 54^o^C, respectively, for 35, 35 and 30 cycles, respectively; *Ihh* and *BSP* primers were used at annealing temperatures of 58^o^C and 54^o^C, respectively, for 30 and 35 cycles, respectively; and *GAPDH* primers were used at an annealing temperature of 55^o^C for 25 cycles. To obtain a semi-quantitative estimate of the relative level of mRNA transcript in cells, the cDNA intensity of the band corresponding to each PCR product was measured by densitometry using ImageJ (NIH software) and normalized to that of the *GAPDH* internal control. In this preliminary study we have used semi-quantitative RT-PCR to evaluate the effects of TRAP on chondrogenic and osteogenic genes, however, quantitative RT-PCR will be considered in the future analyses where more detailed studies will be carried out to delineate molecular mechanisms (i.e., regulation of *WNT3A*, *WNT5A*, *RUNX2*, *MMP-13* and *COL10A1*).

**Statistical analyses**

The sGAG and ALP quantification and PCR data are presented as the mean ± standard error (based on a total of three measurements from three patients [one measurement per patient]) after normalization of data to readouts from control conditions, defined as 1.0, unless otherwise stated. Multi-way ANOVA was carried out along with Bonferroni corrections for multiple comparisons (SPSS 18.0 software).
